# Psychosocial predictors of non-adherence to antihypertensive medication among Korean adults: A nationwide cross-sectional study

**DOI:** 10.1371/journal.pone.0345509

**Published:** 2026-03-30

**Authors:** Kunhee Han, Hwa Sun Kim, Young Lee, Chung-woo Lee

**Affiliations:** 1 Department of Family Medicine, Nowon Eulji Medical Center, Eulji University School of Medicine, Seoul, Republic of Korea; 2 Department of Family Medicine, Veterans Health Service Medical Center, Seoul, Republic of Korea; 3 Veterans Medical Research Institute, Veterans Health Service Medical Center, Seoul, Republic of Korea; Kandahar University, Faculty of Medicine, AFGHANISTAN

## Abstract

The objective of this study was to examine whether depressive symptoms and social vulnerability factors—including living alone, Basic Livelihood Security Program recipient status, and lack of private health insurance—are associated with non-adherence to antihypertensive medication among Korean adults. This cross-sectional analysis used nationally representative data from the Korea National Health and Nutrition Examination Survey (KNHANES), specifically the 2014 (6th cycle–2nd year), 2016 (7th–1st year), 2018 (7th–3rd year), and 2020 (8th–2nd year) cycles, the only survey years that included both depressive symptoms data and antihypertensive medication use information. Non-adherence was defined as taking prescribed medication on fewer than 20 days during the previous month. Depressive symptoms were assessed using the Patient Health Questionnaire-9. Complex survey logistic regression models were used to evaluate associations between psychosocial factors and non-adherence. Two analytic models were applied: individual models for each psychosocial variable and a combined model adjusting for age, sex, obesity, smoking, alcohol use, physical activity, diabetes, and dyslipidemia. Subgroup analyses were performed by age (<70 and ≥70 years). Among adults aged 40 years or older taking antihypertensive medication, approximately one in ten reported non-adherence. In the fully adjusted model, none of the psychosocial factors—including depressive symptoms, living alone, Basic Livelihood Security Program recipient status, or lack of private insurance—were significantly associated with non-adherence in the overall population. However, depressive symptoms were significantly associated with non-adherence among adults aged 70 years or older, whereas no significant associations were observed in younger adults or sex-specific subgroups. These findings suggest that depressive symptoms do not uniformly influence antihypertensive medication adherence across the adult population but may play a more important role in older adults. Incorporating routine screening for depressive symptoms into hypertension management for older individuals may support more effective adherence and improved clinical outcomes.

## Introduction

Hypertension is one of the most common chronic conditions worldwide and a major risk factor for cardiovascular disease [1]. Optimal blood pressure control in patients with hypertension requires consistent adherence to antihypertensive medications [2]. However, studies have reported that approximately 30–50% of individuals with hypertension do not regularly take their prescribed medications [3], and such non-adherence is associated with poor treatment outcomes and an increased risk of cardiovascular events [4]. Recent large-scale studies reaffirm that medication non-adherence remains a major barrier to hypertension control, with psychosocial factors such as depressive symptoms increasingly recognized as key determinants [5].

Factors influencing adherence to antihypertensive medications are multifaceted. In addition to structural barriers, such as cognitive impairment, limited disease awareness, and financial constraints, psychosocial factors, particularly depressive symptoms, have been identified as important contributors [6,7]. Depressive symptoms can negatively affect medication-taking behavior by reducing motivation, inducing fatigue, and impairing cognitive function [8,9]. Nevertheless, several previous studies focused on patients with a formal diagnosis of depression, whereas the influence of subclinical depressive symptoms remains relatively underexplored [5,10,11]. A systematic review published in 2024 demonstrated a consistent association between depressive symptoms and antihypertensive medication non-adherence across diverse populations [11].

Recently, growing attention has been paid to the effects of social isolation and socioeconomic vulnerability on health-related behaviors, particularly in the context of increasing numbers of single-person households and aging population [12, 13]. Social vulnerability factors, such as living alone or economic hardship, may indirectly hinder consistent health-promoting behaviors, including medication adherence [14]. However, few studies have directly compared the relative impact of structural and emotional factors on adherence among individuals with hypertension.

This study aimed to investigate the relationship between social vulnerability factors and non-adherence to antihypertensive medications, with particular focus on depressive symptoms in a Korean adult population. By including individuals who had not been clinically diagnosed with depression but were positive for depressive symptoms using the Patient Health Questionnaire-9 (PHQ-9) [15], we sought to examine a high-risk group frequently encountered in primary care that is often overlooked. Additionally, subgroup analyses according to age were conducted to further characterize individuals at an increased risk of non-adherence.

## Methods

### Study design and data source

This study employed a cross-sectional design using data from the Korea National Health and Nutrition Examination Survey (KNHANES), which is a nationally representative survey conducted by the Korea Disease Control and Prevention Agency. We used data from the 2014 (6th cycle–2nd year), 2016 (7th–1st year), 2018 (7th–3rd year), and 2020 (8th–2nd year) KNHANES cycles. These four years were specifically selected because they were the only survey years that simultaneously included both PHQ-9 depressive symptoms data and information on antihypertensive medication use. The KNHANES uses a stratified multistage probability sampling method to select households and collect data through health interviews, physical examinations, and nutrition surveys [16].

### Study population

We included participants aged 40 years and older who reported a diagnosis of hypertension and current use of antihypertensive medication, as hypertension prevalence and medication use are markedly higher from middle age onward, and the number of treated cases under age 40 was too small for stable statistical analysis. Hypertension was defined as systolic blood pressure of ≥140 mmHg, diastolic blood pressure of ≥90 mmHg, or a previous medical diagnosis of hypertension. The participants were excluded if they had been diagnosed with depression, were currently taking antidepressants, or had missing data on key study variables. The study size was determined by the number of eligible participants meeting these inclusion criteria in the KNHANES dataset, rather than by a priori sample size calculation.

### Key variables

#### Non-adherence to antihypertensive medication.

Medication adherence was assessed using self-report. Participants who reported taking antihypertensive medication for <20 days in the past month were classified as non-adherent. We defined antihypertensive medication non-adherence as self-reported intake on fewer than 20 days per month, consistent with previous Korean studies using KNHANES data [17,18]. This cutoff reflects a practical threshold commonly used in Korean population-based surveys, including the Korea National Health and Nutrition Examination Survey (KNHANES), in which antihypertensive treatment status is operationally defined as medication use on ≥20 days per month to reflect meaningful treatment engagement in daily life [19]. Although a direct correspondence between monthly days of medication use and the validated multi-item adherence scales such as the MMAS-8 scores is not established, taking antihypertensive medications on fewer than 20 days per month is conceptually consistent with the low-adherence category of the MMAS-8 [20].

#### Psychosocial factors.

Depressive symptoms were assessed using the full nine-item PHQ-9, available in KNHANES 2014, 2016, 2018, and 2020. Each item evaluates the frequency of specific depressive experiences during the past two weeks on a four-point scale (0 = not at all to 3 = nearly every day). Item scores were summed to yield a total score ranging from 0 to 27, and participants with a score ≥ 10 were classified as having depressive symptoms [15, 21]. Social vulnerability indicators included living alone (defined as self-reported single-person households) [22], receiving benefits from the Basic Livelihood Security Program (BLSP), and lack of private health insurance [23].

#### Covariates.

Demographic and health-related variables included sex, age (continuous and dichotomized at 70 years), obesity (body mass index ≥25 kg/m²), abdominal obesity (based on waist circumference and sex-specific cut-offs), current smoking, alcohol use (≥1 time/month), physical activity (≥150 min/week of moderate or ≥90 min/week of vigorous activity), diabetes mellitus (defined by HbA1c of ≥6.5% or diagnosis/treatment), and dyslipidemia (based on total cholesterol levels, diagnosis, or treatment).

### Statistical analysis

All analyses were conducted using complex sample analysis methods to account for the multistage stratified survey design and sampling weights. Baseline characteristics were calculated using survey weights, stratification, and clustering to reflect the complex sampling design of KNHANES.

Multivariable logistic regression analyses were performed to examine the independent associations of depressive symptoms, living alone, BLSP recipient status, and private health insurance with antihypertensive medication non-adherence. Two analytic models were applied: (1) separate models including each psychosocial factor individually, and (2) a combined model including all four psychosocial variables simultaneously, adjusting for age, sex, obesity, smoking, alcohol use, physical activity, diabetes mellitus, and dyslipidemia.

Both unadjusted and adjusted odds ratios (ORs) with 95% confidence intervals (CIs) were estimated. Subgroup analyses were performed according to age group (<70 and ≥70 years).

To minimize potential sources of bias, we applied sampling weights to account for the complex, multistage survey design and to ensure representativeness of the Korean adult population. Participants with missing data on key variables were excluded to avoid information bias, and all models were adjusted for relevant sociodemographic and clinical covariates to mitigate confounding effects.

All analyses were performed using R software (version 3.6; R Foundation for Statistical Computing, Vienna, Austria).

No artificial intelligence–based tools were used during the study period.

### Ethical considerations

This study was reviewed and approved by the Institutional Review Board of the Veterans Health Service Medical Center (BOHUN 2025-01-011-003). We accessed the KNHANES datasets for research purposes on 11 December 2024. Because the data were publicly available and anonymized, informed consent from the participants was waived. The authors did not have access to any information that could identify individual participants during or after data collection.

## Results

### Baseline characteristics

A total of 6,485 survey-weighted adults aged ≥40 years with hypertension and current antihypertensive medication use were included. Table 1 presents the survey-weighted baseline characteristics of the study population. Overall, 46.1% were female and 28.9% were aged ≥70 years. Regarding clinical factors, 27.6% had diabetes, 33.9% had dyslipidemia, and 66.7% reported alcohol use, while 95.7% did not meet recommended levels of physical activity. With respect to psychosocial characteristics, 5.0% of participants had depressive symptoms (PHQ-9 ≥ 10), 14.3% lived alone, 7.5% were recipients of the BLSP, and 31.3% lacked private health insurance.

**Table 1:** Baseline characteristics of the study population.

Variable	Total (n = 6,485)
Female	2,990 (46.1%)
Age ≥ 70	1,874 (28.9%)
Type 2 diabetes	1,790 (27.6%)
Dyslipidemia	2,198 (33.9%)
Alcohol use (Yes)	4,325 (66.7%)
Physical inactivity	6,206 (95.7%)
Depressive symptoms	324 (5.0%)
Living alone	927 (14.3%)
BLSP recipient	486 (7.5%)
No private insurance	2,434 (31.3%)

Abbreviations: BLSP, Basic Livelihood Security Program.

Note: Values are presented as number (percentage).

### Association between psychosocial factors and medication non-adherence

Table 2 presents the results of the survey-weighted combined multivariable logistic regression model for the overall study population. In the fully adjusted model, none of the psychosocial variables were significantly associated with antihypertensive medication non-adherence. Depressive symptoms showed a non-significant trend toward higher odds of non-adherence (adjusted OR = 1.50, 95% CI 0.94–2.40, p = 0.092). Living alone (aOR = 1.22, 95% CI 0.85–1.75, p = 0.275) and BLSP recipient status (aOR = 1.12, 95% CI 0.67–1.85, p = 0.668) were not associated with non-adherence. Lack of private health insurance was also not significantly associated with non-adherence (aOR = 0.81, 95% CI 0.59–1.10, p = 0.172). All estimates were derived from survey-weighted logistic regression models accounting for KNHANES sampling weights, stratification, and clustering.

**Table 2:** Survey-weighted logistic regression models examining psychosocial factors associated with antihypertensive medication non-adherence.

Variable	Model 1: Individual model		Model 2: Combined model	
	Adjusted OR (95% CI)	p-value	Adjusted OR (95% CI)	p-value
Depressive symptoms	1.58 (1.02-2.45)^*^	0.041	1.50 (0.94–2.40)	0.092
Living alone	1.29 (0.92-1.82)	0.139	1.22 (0.85–1.75)	0.275
BLSP recipient	1.18 (0.73-1.92)	0.495	1.12 (0.67–1.85)	0.668
No private insurance	0.85 (0.63-1.15)	0.293	0.81 (0.59–1.10)	0.172

Abbreviations: BLSP, Basic Livelihood Security Program.

Adjusted for age, sex, obesity, smoking, alcohol use, physical activity, diabetes mellitus, and dyslipidemia.

All models accounted for KNHANES sampling weights, stratification, and clustering.

Model 1: Each psychosocial factor analyzed separately and adjusted for covariates.

Model 2: All psychosocial variables analyzed simultaneously with covariate adjustment.

* Statistically significant.

### Subgroup analysis by age (<70 and ≥70 years)

As shown in Table 3, depressive symptoms were significantly associated with antihypertensive medication non-adherence among adults aged ≥70 years (aOR = 2.54, 95% CI 1.30–4.95, p = 0.006), whereas no significant association was observed among those aged <70 years (aOR = 1.13, 95% CI 0.58–2.23, p = 0.716). However, formal tests for interaction between depressive symptoms and age group were not statistically significant in either the combined model or the individual models (all P for interaction > 0.05), indicating that there was no statistical evidence that the strength of the association differed by age.

**Table 3:** Survey-weighted combined multivariable logistic regression model examining psychosocial factors associated with antihypertensive medication non-adherence by age group (<70 and ≥70 years).

Variables	Age < 70 years		Age ≥ 70 years	
	Adjusted OR (95% CI)	p-value	Adjusted OR (95% CI)	p-value
Depressive symptoms	1.13 (0.58-2.23)	0.716	2.54 (1.30–4.95)^*^	0.006
Living alone	1.27 (0.77-2.10)	0.351	1.38 (0.83–2.29)	0.219
BLSP recipient	0.96 (0.52-1.80)	0.906	1.59 (0.76–3.32)	0.217
No private insurance	0.89 (0.58-1.35)	0.570	0.72 (0.42–1.23)	0.233

Abbreviations: BLSP, Basic Livelihood Security Program.

Adjusted for age, sex, obesity, smoking, alcohol use, physical activity, diabetes mellitus, and dyslipidemia.

All models accounted for KNHANES sampling weights, stratification, and clustering. All psychosocial variables analyzed simultaneously with covariate adjustment.

* Statistically significant.

This suggests that depressive symptoms may have a stronger behavioral impact among older adults, potentially reflecting age-related cognitive, functional, or motivational vulnerabilities that affect medication-taking routines.

Across both age groups, none of the social vulnerability indicators—living alone, BLSP recipient status, or lack of private health insurance—were significantly associated with non-adherence in the fully adjusted models. Although point estimates for living alone and BLSP recipient status were higher among adults aged ≥70 years, interaction tests were not statistically significant, suggesting no clear age-dependent modification of these associations. These results indicate that emotional vulnerability, rather than socioeconomic or structural disadvantage, may play a more prominent role in medication adherence among older individuals.

## Discussion

In the overall population, depressive symptoms were not significantly associated with antihypertensive medication non-adherence in the fully adjusted model. Although previous studies have frequently reported a positive association between depressive symptoms and poor adherence in hypertensive adults [5–10], many of these analyses were conducted in clinical cohorts or high-risk subgroups with greater psychiatric comorbidity. Our findings suggest that, within a nationally representative sample of community-dwelling adults, depressive symptoms alone may not be a uniform or dominant determinant of medication-taking behavior. This implies that the influence of depressive symptoms on adherence may be context-dependent and shaped by population characteristics, health system factors, or the severity of emotional distress.

Unlike many earlier studies that identified a significant association between depressive symptoms and poor medication adherence, our analysis did not demonstrate a statistically significant relationship in the overall sample. This discrepancy may reflect differences in population characteristics, measurement approaches, or cultural factors that influence how depressive symptoms translate into self-management behaviors [6,24]. While prior research—largely conducted in Western populations—reported a consistent association in general hypertensive cohorts, our findings suggest that this relationship may be more context-dependent or moderated by age. Depressive symptoms appeared to influence adherence predominantly among older adults in our Korean cohort, indicating that cultural norms, health system characteristics, or age-related psychosocial vulnerabilities may alter the extent to which depressive symptoms affect medication-taking behavior.

The particularly strong association observed in older adults may be explained by several interrelated mechanisms. Older adults are more likely to experience cognitive decline, polypharmacy, lower health literacy, and functional limitations, all of which can impede adherence [24,25]. Depressive symptoms may exacerbate these barriers by reducing motivation, impairing executive function, or increasing the perceived burden of treatment [26, 27]. Additionally, the co-occurrence of social isolation, low income, and health system barriers among older adults can further compound the effects of depressive symptoms on self-care behaviors [12].

Beyond these contextual factors, several psychological mechanisms may link subclinical depressive symptoms to non-adherence. First, motivational deficits and anhedonia can diminish the willingness to engage in daily self-care tasks, including medication-taking routines. Second, mild executive dysfunction—characterized by impaired planning, concentration, and working memory—may hinder consistent medication management and reduce the ability to organize health behaviors effectively. Third, social withdrawal and reduced help-seeking behaviors commonly associated with depressive symptoms can limit access to social or instrumental support that might otherwise reinforce adherence [8,27]. Together, these cognitive and behavioral mechanisms likely mediate the pathway between emotional distress and non-adherence, explaining why even subthreshold depressive symptoms can have clinically meaningful effects on treatment behavior, particularly in older adults.

Interestingly, traditional structural and socioeconomic factors, such as living alone, being a recipient of the BLSP, or lacking private health insurance, were not significantly associated with non-adherence in the adjusted models. This suggests that emotional health may have a more proximal and potent influence on medication behavior than structural disadvantages, at least in this population. However, in the stratified analyses, living alone and being a recipient of public assistance showed borderline associations, with non-adherence among older adults, suggesting that psychosocial vulnerabilities may have cumulative effects in this subgroup. These findings are in line with prior studies showing that perceived social support, rather than objective indicators, such as marital or cohabitation status, is more closely related to medication non-adherence [28, 29].

From the clinical and public health perspectives, these results underscore the importance of incorporating mental health screening into chronic disease management, particularly in primary care settings [30,31]. The use of brief screening tools, such as the PHQ-9, can help identify patients with elevated depressive symptoms who may be at increased risk of poor adherence [32]. Moreover, the finding that even undiagnosed depressive symptoms affect medication-taking behavior supports the need for early low-intensity interventions targeting emotional distress in hypertensive populations.

In addition to screening, multifaceted interventions that address both psychological and structural barriers may be particularly effective in older adults [33,34]. These may include simplification of medication regimen, community-based adherence support, and interventions to improve health literacy and self-efficacy [29, 35, 36]. The integration of such strategies into team-based primary care or community health services may be particularly beneficial for socially vulnerable older individuals [37].

This study has several notable strengths. First, it utilized data from a nationally representative population-based survey with rigorous sampling methods, thereby enhancing generalizability. Second, depressive symptoms were assessed using the validated PHQ-9 instrument, which allows standardized and quantifiable measurements of subclinical mood disturbances. Third, the analysis was controlled for a wide range of potential confounders and included stratified analyses according to age and sex, allowing for the exploration of effect modification. Finally, the simultaneous examination of psychological and socioeconomic determinants of non-adherence provides a more comprehensive understanding of behavioral risk factors in hypertension management.

Despite these strengths, this study has certain limitations. First, the cross-sectional design limited the ability to infer causal relationships between depressive symptoms and medication non-adherence. Second, both depressive symptoms and medication non-adherence were assessed using self-reported measures, which are susceptible to recall and social desirability biases. Third, participants with clinically diagnosed depression were excluded to isolate the impact of subclinical symptoms; however, this may cause selection bias and limit the generalizability of the findings to individuals with more severe affective disorders. Fourth, the assessment of medication non-adherence was based on self-reported recall of antihypertensive medication use during the past month, using a cutoff of fewer than 20 days. This operational definition follows the approach used in prior KNHANES-based studies, which have similarly categorized participants taking antihypertensive medication on fewer than 20 days per month as non-adherent [17,18]. Although this cutoff has practical value and allows for comparability within the Korean national survey framework, it has not been formally validated against established adherence measures such as the Morisky Medication Adherence Scale or prescription refill data [38]. Consequently, some degree of measurement error and limited comparability with studies using validated adherence tools cannot be ruled out. Finally, the evaluation of social vulnerability was limited to binary indicators, such as living alone, receiving income support, and private insurance coverage, which may not fully capture the complexity of psychosocial risk factors. Constructs, such as perceived social support, loneliness, health literacy, and self-efficacy, were not available in the dataset; therefore, they were not considered in the analysis.

It should also be noted that part of the study data was collected in 2020, during the COVID-19 pandemic. The pandemic period was associated with increased psychological distress, social isolation, and disruptions in healthcare utilization, all of which could have influenced both depressive symptoms and medication non-adherence behaviors [39–41]. Although our analyses adjusted for survey year, residual effects related to the pandemic context cannot be completely ruled out. Future research using longitudinal data will be needed to clarify the long-term impact of such environmental stressors on mental health and treatment adherence.

Future studies should employ longitudinal designs to assess the temporal relationship between depressive symptoms and medication adherence, as well as explore causal mechanisms through mediation analysis. Intervention studies targeting mood symptoms, particularly in older adults with hypertension, are warranted. Moreover, the inclusion of comprehensive psychosocial variables, such as loneliness, cognitive function, and self-efficacy, would further elucidate the pathways through which depression influences non-adherence.

These findings highlight the importance of integrating mental health assessment into routine hypertension management, particularly for older adults. The coexistence of depressive symptoms and poor medication adherence suggests that collaborative care models linking primary care and mental health services may improve treatment outcomes. In both clinical and community settings, brief validated screening tools such as the PHQ-9 could be used to identify individuals at risk for non-adherence. Moreover, interventions that combine psychological support with strategies to simplify medication regimens and enhance self-efficacy should be considered in chronic disease management programs for older adults.
